# Comments on *A new theory for X-ray diffraction*

**DOI:** 10.1107/S2053273318003959

**Published:** 2018-07-18

**Authors:** Jack T. Fraser, Justin S. Wark

**Affiliations:** a Department of Physics, Clarendon Laboratory, Parks Road, University of Oxford, Oxford, OX1 3PU, England

**Keywords:** diffraction theory, powder diffraction, small crystals

## Abstract

In an article entitled *A new theory for X-ray diffraction* [Fewster (2014 ▸). *Acta Cryst.* A**70**, 257–282], hereafter referred to as NTXRD, it is claimed that when X-rays are scattered from a small crystallite, whatever its size and shape, the diffraction pattern will contain enhanced scattering at angles of exactly 2θ_B_, whatever the orientation of the crystal. It is claimed that in this way scattering from a powder, with randomly oriented crystals, gives rise to Bragg scattering even if the Bragg condition is never satisfied by an individual crystallite. The claims of the theory put forward in NTXRD are examined and they are found to be in error. Whilst for a certain restricted set of shapes of crystals it is possible to obtain some diffraction close to (but not exactly at) the Bragg angle as the crystallite is oriented away from the Bragg condition, this is generally not the case. Furthermore, contrary to the claims made within NTXRD, the recognition of the origin of the type of effects described is not new, and has been known since the earliest days of X-ray diffraction.

## Abstract 2

Fewster [(2014), *Acta Cryst.* A**70**, 257–282] claimed that a new theory of X-ray diffraction is required, and that small crystallites will give rise to scattering at angles of exactly twice the Bragg angle, whatever their orientation. This article demonstrates that this theory is in error.

## 1. Introduction   

Despite the field of X-ray diffraction being more than a century old, in an article entitled *A new theory for X-ray diffraction* (Fewster, 2014 ▸), hereafter referred to as NTXRD, it is claimed that a new theory of diffraction is required to explain the intensities observed in powder diffraction and other diffraction geometries. Within NTXRD a theory of X-ray diffraction is proposed which predicts that ‘*the scattering from a crystal or crystallite is distributed throughout space [which] leads to the effect that enhanced scatter can be observed at the ‘Bragg position’ even if the ‘Bragg condition’ is not satisfied*’ and that ‘*the scatter from a single crystal or crystallite, in any fixed orientation, has the fascinating property of contributing simultaneously to many ‘Bragg positions’*’. If this new approach were correct it would certainly have significant implications for the whole field of X-ray diffraction, and given the prominence afforded to this new theory (it featured on the front cover of the published volume), its veracity or otherwise deserves appropriate scrutiny. However, we show here that the analysis presented within NTXRD is incorrect, and that the underlying concepts upon which the theory is based are not new but were known to the earliest pioneers of X-ray diffraction.

At the outset we emphasize that in this article we will not ourselves be undertaking the task of proposing an explanation for the several interesting pieces of experimental data presented by Fewster, which are certainly worthy of further study and attention. Rather, our more restricted aim is to demonstrate that the new theory that he puts forward is incorrect, and we identify the sources of error in the arguments put forward in NTXRD. Secondly, whilst the interested reader would no doubt benefit from reading the NTXRD article in full, we present in the section below the key result of the theory within NTXRD which we deem to be erroneous. Thirdly, it is important to note that the new theory of X-ray diffraction that Fewster puts forward is based on a set of highly simplifying assumptions. These assumptions are the very same approximations made over a century ago by the doyens of the field. We adopt the same approach here: following Fewster we will be assuming that the crystal of interest is irradiated by a monochromatic plane wave with a transverse coherence length larger than the crystal, and that the diffraction observed in the far field is in the Fraunhofer limit: that is to say that the size of the illuminated crystal is 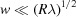, where *R* is the distance to the detector and λ the wavelength of the X-rays, such that the condition should be reasonably well obeyed for diffraction from crystals of the order of 1 µm in size when the detector is several tens of cm distant. Further, the kinematic approximation with zero absorption is also assumed, we treat the atoms as point scatterers, and neglect the effects of polarization and of finite temperature. Whilst it is well known that the assumptions made above can break down even for diffraction from small crystallites (Shabalin *et al.*, 2017 ▸), for the sake of direct comparison we use the same assumptions as those made in NTXRD.

## 2. Fewster’s theory   

**Figure 1:**
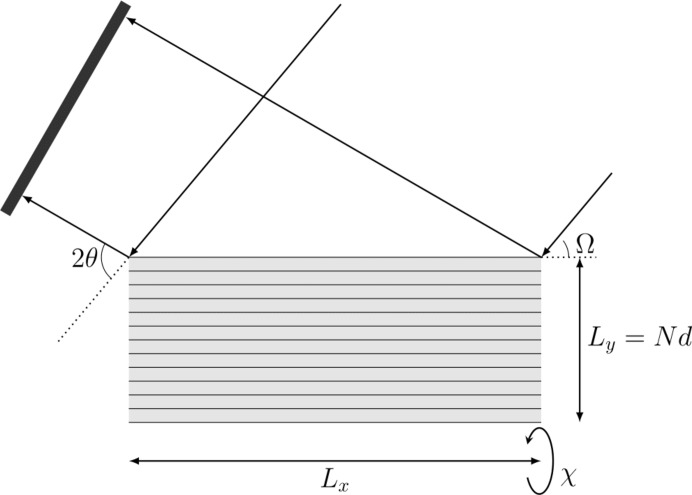
A schematic diagram of the diffraction setup. Radiation is incident on a crystal plane at a variable angle Ω, and the detector is placed at an angle of 2θ with respect to the incident X-rays. χ denotes the rotation axis used in §7.

Consider the diffraction geometry shown in Fig. 1 ▸, adapted from Fig. 4(*a*) of NTXRD. Fewster derives the following formula [the square of the amplitude, 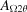, calculated in equation (5) of NTXRD] for the scattered intensity from a set of atoms, recorded by a detector placed at an angle 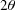 to a beam of monochromatic radiation of wavelength λ which is incident at an angle Ω to the crystal plane: 
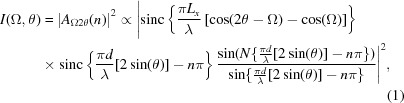
where 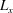 is the length of the crystal, *d* is the plane spacing, *n* denotes the ‘order’ of planes from which the X-rays are diffracting and *N* is the number of planes in the stack contributing to the reflection.

This formula predicts maxima in the scattered intensity whenever 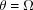 (*i.e.* a specular peak) and when 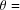 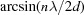 (the Bragg peak), no matter what angle the crystal is placed at relative to the incident beam, and this prediction forms the fundamental basis of the new theory of diffraction described in NTXRD. However, equation (1) is incorrect and, as we shall show, the actual formula for the angle-dependent scattering, known since the earliest days of X-ray diffraction, leads to substantially different conclusions. We discuss the error in Fewster’s analysis in §3, after first outlining the specific predictions of NTXRD.

**Figure 2:**
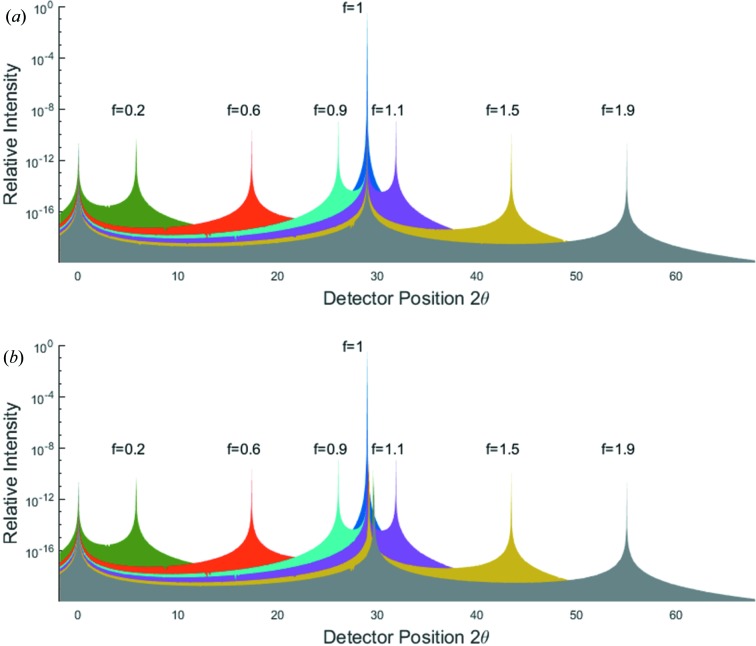
A comparison of the predictions of (*a*) equation (1), the NTXRD result for 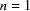, and (*b*) equation (21), our result for a cubic shaped crystallite with faces aligned to the cube axes, for radiation incident at an angle 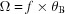 for a variety of *f* values. Both distributions exhibit specular reflections. Whilst the NTXRD result predicts a further peak at exactly 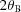, we find this second peak to vary in angle as described in the text.

In Figs. 2 ▸(*a*) and 3 ▸(*a*) we plot the intensity observed at 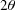, calculated from 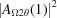 and 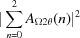, respectively, where 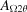 is defined as in equation (1), for a range of angles of incidence, 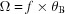. For this particular case we have set 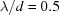 and 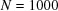 [this ratio of wavelength to spacing is within 2% of that used by Fewster, who uses a value of 0.491 corresponding to the diffraction of Cu *K*α radiation from the (111) planes of silicon, although specific lattices are not mentioned within NTXRD]. It can be seen in both figures that for all values of Ω there is some enhanced scattering at a position corresponding to exactly that of the Bragg condition, along with a peak that corresponds to specular scattering (the two being identical for 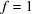).

Note that the inclusion of the planes 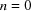 and 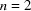 makes Fig. 2 ▸(*a*) identical to Fig. 5 of NTXRD. On the basis of this plot it is claimed within NTXRD that for a set of crystallites with random orientations the specular scattering associated with each crystal will occur at different scattering angles, thus producing a background intensity, whereas because each crystallite produces some scattering at exactly the Bragg condition, the intensities at the scattering angle 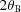 from all of the crystals add, giving rise to a sharp peak. This result forms the basis of the work within NTXRD. However, we show below this analysis to be in error.

## 3. The error in Fewster’s analysis   

Fewster’s analysis contains three errors – one minor and two major. Firstly, he states that the amplitude, 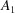, of X-rays diffracted from a single (the first) plane shown in Fig. 1 ▸ is given by 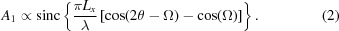
This is clearly the scattering amplitude from a uniform plane. However, if instead we consider scattering from *N* discrete atoms (assumed here to be point-like, *i.e.* ignoring the atomic form factor) separated by a distance *a*, the scattered amplitude from a single plane of atoms is 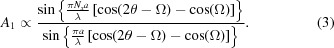
This is only a minor error since, in the small-angle limit, equations (2) and (3) are in very close agreement, but diverge for larger angles (we discuss further the relationship between the use of sinc functions to describe the diffraction and the ratio of two sine functions in §4).

The first of the major errors in Fewster’s analysis is as follows. He states correctly that the phase difference for the scattering from successive planes, 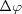, is 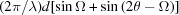. However, he erroneously assumes that this phase difference can be approximated as 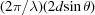. This is incorrect, and it is this approximation that leads to NTXRD always giving a peak in the scattered intensity at the Bragg condition. We discuss the origin of this approximation below. If one instead uses the correct phase difference, then summing the complex amplitudes over the 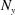 planes yields 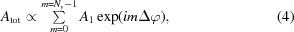
which, on inserting the correct value of 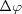, yields 
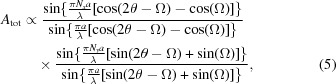
where we have set 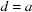 (a simple cubic lattice) and which can be recognized as the two-dimensional form of the result obtained by Scherrer (1918 ▸), as outlined in the classic text by Warren (1969 ▸).

A further error asserted within NTXRD is that the analysis presented holds true for all crystal shapes. This is incorrect. The results presented in this section only hold true for an orthorhombic crystal with the sides cut parallel to the unit-cell axes. We discuss diffraction from more general crystal shapes in §5.

At this juncture we discuss in more depth the origin of the specific error leading Fewster to assert that some peak in the scattered intensity always occurs at the Bragg condition, independent of Ω. Whilst we find it somewhat difficult to follow the line of reasoning taken in NTXRD (as it appears to rely on only taking into account specific scattering points, rather than correctly summing all of the complex amplitudes from all scatterers), during the preparation of this article Paul Fewster drew our attention to one of his later articles within which he puts forward additional arguments as to why he maintains there is always enhanced scattering at the Bragg condition (Fewster, 2016 ▸). However, whilst the additional argument within Fewster (2016 ▸) is also deeply flawed, it does give some further insight into the origin of the error. Consider the diagram shown in Fig. 4 ▸ [adapted from Fewster (2016 ▸)], which shows the path length 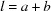 between two points (where here *a* denotes the distance shown in Fig. 4 ▸, rather than the lattice spacing): the first, 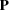, in the upper plane, and a point 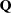 in the lower plane. As pointed out by Fewster, the difference in path length between the waves scattering from 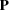 and 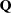 is given by 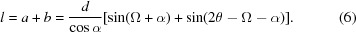
The nub of the claim in Fewster (2016 ▸) is that it can be shown that for a fixed scattering point, 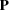, the relevant number of scattering points, 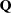, in the next plane that scatter with a path length that differs from λ by 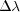 (where 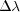 is some fixed difference in path length that we choose such that 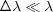) maximizes at the Bragg condition 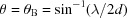 *independent of* Ω, and hence enhanced scattering will always be seen at the Bragg angle. This is illustrated in Fig. 3 of Fewster (2016 ▸), which we shall in due course replicate below. We assume that this is why, in Fewster (2014 ▸), he makes the small-angle approximation detailed above. However, we demonstrate below that the above claim is also false and identify the origin of the error.

Let us consider how we *should* calculate the effective length, 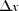, along the lower plane that contains points that scatter in such a way so as to have a path length with respect to 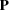 that differs from λ by 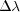 (as the relevant scattering amplitude will be proportional to this length). Let the coordinate of 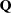 along its plane be *x* (such that the fixed *x* coordinate of 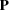 is 0). Then, the length along the lower plane that contains points that scatter with path lengths within 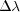 of λ will be proportional to 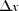, 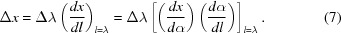



We state here the error that Fewster makes. He does not calculate the number density of points in the second plane as a function of the deviation in the path difference. Instead, he calculates the number density of path lengths that are within 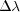 of λ as a function of α, and then evaluates how many scattering points in the second plane are associated with each of the path lengths that fulfil this condition. To put it in simple mathematical terms, he effectively only considers the second term appearing in the chain rule on the right-hand side of equation (7), *i.e.* he erroneously assumes 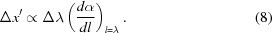



**Figure 3:**
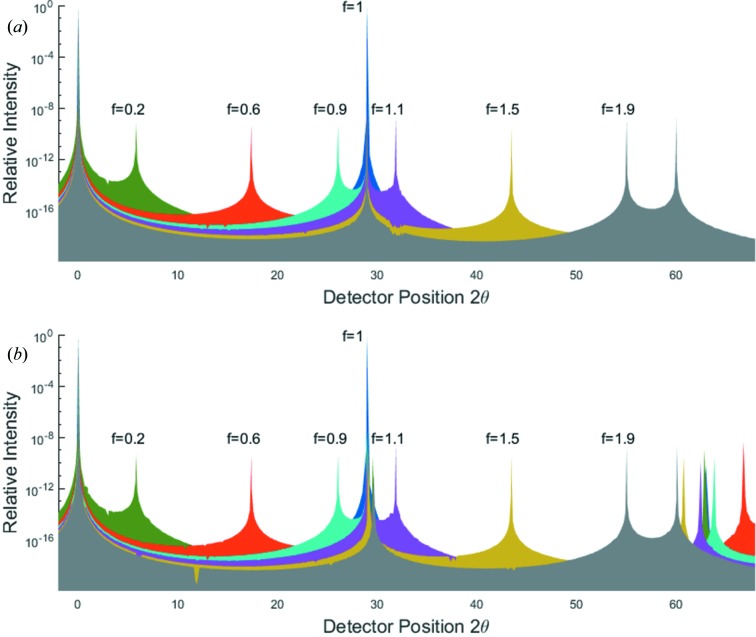
A comparison of the predictions of the theory presented in (*a*) NTXRD and in (*b*) the theory represented by equation (20), including contributions from higher-order planes. In addition to the deviations previously noted in Fig. 2 ▸, the higher-order terms of equation (20) produce subsidiary maxima in (*b*) which are not present in (*a*).

**Figure 4:**
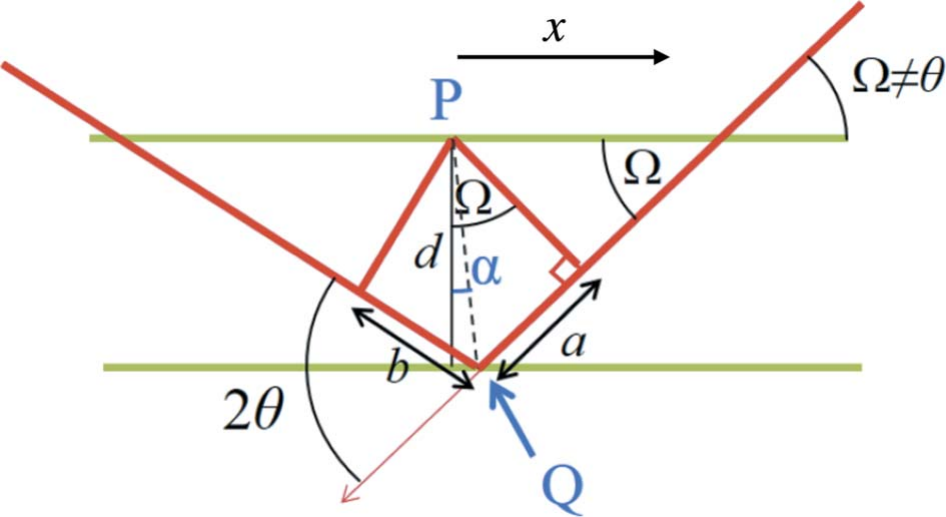
The geometry for calculating the positions 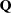, in terms of incident angles and detector capture angles 2θ, used to construct the path lengths described by equation (6): *l* = *a* + *b* = 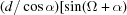 + 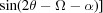 [adapted from Fewster (2016 ▸)].

That this is being assumed can be confirmed by examining the short Python code in the supporting information to Fewster (2016 ▸), from which Fig. 3 in that article is produced, and from the statement within Fewster (2016 ▸) that ‘*we can decide on an acceptable path difference*, 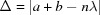 *and sum the number of* α *values, for specific* Ω *and* 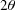 *values, that have a path difference* 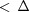
’. Whilst this term does indeed peak close to the Bragg angle for all Ω, it does not represent the required physical quantity which is correctly described by equation (7). When multiplied by the second term in the chain rule [*i.e.* equation (7) is evaluated], this effect vanishes, as would be expected. Let us now show this. From equation (6) differentiation of *l* with respect to α yields 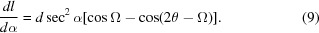
However, to evaluate equation (8) we seek solutions where the 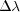 is the deviation in path length from λ. Now, by rearrangement of equation (6) with 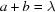


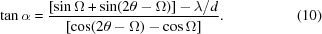
Substituting this solution for 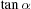 (with 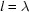) from equation (10) into equation (9) we find 
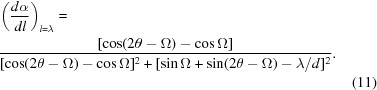



**Figure 5:**
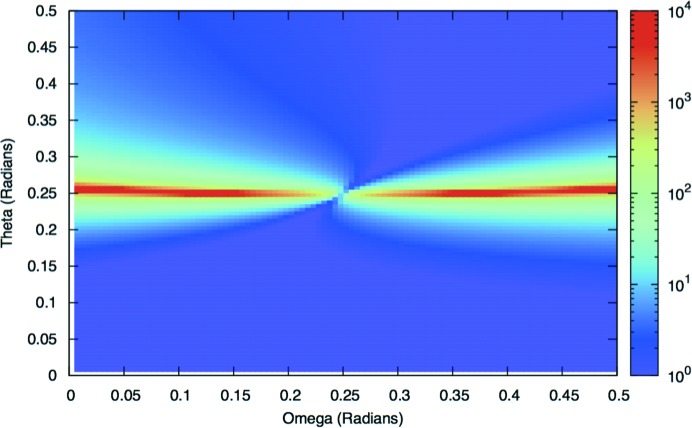
A plot of 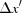 as a function of θ and Ω as calculated from equation (8). Note this is identical in form to Fig. 3 in Fewster (2016 ▸).

We plot 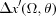 calculated from equations (8) and (11) in Fig. 5 ▸, using the same ratio of λ and *d* (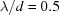) as used in Fewster (2016 ▸). It can be seen that whilst equation (11) does not appear in Fewster (2016 ▸), this plot is indeed identical in form to Fig. 3 in Fewster (2016 ▸). We stress again that this does not represent the relevant number of points along the scattering plane that scatter with path lengths within 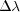 of λ. That density is represented by equation (7), which we now evaluate.

Consider the first term in the chain rule of equation (7). As 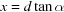, then, without setting the constraint 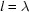, 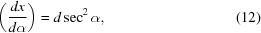
and substituting equations (9) and (12) into equation (7) we find 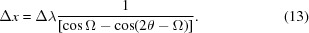
We note that this is a function with no dependence on α. Since in the derivation above we have not yet set the constraint 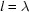, a large value of 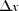 represents a turning point in the path difference of any value, 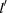. From equation (13), we see that 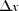 always maximizes upon the specular condition being met (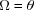) (or conversely we can say that the path length as a function of the position of 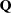 minimizes at this condition), and thus for 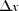 to maximize under the constraint that 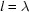, then 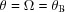, consistent with traditional diffraction theory. As expected, no scattering peak at 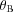 is seen at any other value of Ω.

## 4. Calculation of the scattered intensity   

In order to elucidate further errors described in NTXRD, in this section we note the well known result that equation (5) can, *via* the method of Poisson sums, be written in terms of the Fourier transform of the shape function of an orthorhombic shaped crystal (sinc functions) centred on the infinite reciprocal lattice [see equation (20) below]. By use of such shape functions we will, in §5, show results for diffraction from spherical crystals, which are also discussed erroneously in NTXRD.

Furthermore, we will demonstrate that some enhancement close to, but not exactly at, the Bragg condition can arise from the conventional analysis of diffraction from certain planes of a restricted set of shapes of crystals, without the need to appeal to a new theory. By working in reciprocal space we illustrate the origin of these ‘Bragg-like’ peaks, as well as of the specularly diffracted radiation, and show that, contrary to the claims within NTXRD, these types of effects are well known.

Under the simplifying assumptions made in §1, the intensity of radiation scattering from a crystal of *N* atoms is given by 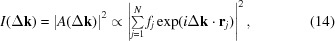
where 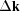 is the difference between the wavevectors of the incident and scattered radiation, 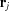 is the position of atom *j* and 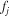 is the usual atomic form factor. In order to calculate the diffraction pattern from a finite crystal of a particular shape, we use the method of Poisson sums in three dimensions (Stein & Weiss, 1971 ▸), which gives, for a well behaved function *g*, 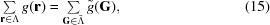
where Λ and 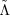 are the direct and reciprocal lattices, respectively, 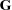 is a reciprocal-lattice vector and 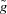 is the three-dimensional Fourier transform of *g*.

We consider first a crystal infinite in extent. By writing 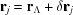, where 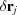 is the relative coordinate of the atom in the basis and 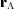 is the position of the associated lattice point, equation (14) can be rewritten as sums over the lattice vectors 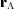 and the basis *B*: 
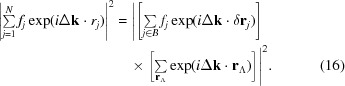



At this stage, assuming that the left-hand side of the above equation extends over an infinite crystal, we can apply equation (15), giving the result that diffraction only occurs when the Bragg condition is satisfied: 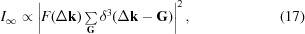
where 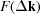 is the geometric structure factor 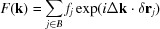.

For a crystal that is finite in extent, the sum can be extended over the infinite lattice Λ by introducing a function, 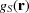, to describe the shape of the crystal, such that 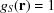 within the volume *S* enclosed by the surface of the crystallite, and 0 elsewhere. Equation (14) can then be written 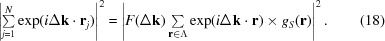
Thus, using equation (15) and the convolution theorem, 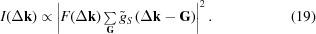
Here 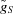 is the three-dimensional Fourier transform of the shape function (‘the shape transform’). In reciprocal space, equation (19) has a simple geometric interpretation: it is the convolution of the shape transform 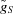 with the reciprocal lattice.

For the purposes of this article, we will be dealing with a single-atom basis and we shall also assume point-like scattering, such that we may assume throughout that 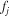 and *F* are independent of 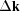. As with the initial analysis of NTXRD, we have also ignored the effects of absorption and extinction.

Consider a crystallite with a primitive cubic lattice of lattice spacing *a*. We assume that the shape of the crystallite is orthorhombic and that the normals to the faces of the cube lie along the principal axes of the cubic unit cell such that the size of the crystallite 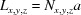. The reciprocal lattice is cubic, with reciprocal-lattice spacing 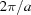, and this is convolved with the shape transform of the crystal such that equation (19) yields 
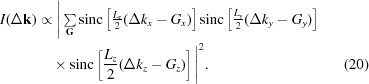
The equation above shows the link between the form used by Scherrer [equation (5), ratios of sine functions] and a set of sinc functions which are functions of 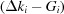, but then summed over all reciprocal-lattice vectors. The two forms produce identical results, but using the approach of equation (19) is more convenient for the present discussion, as it allows us readily to calculate the diffracted intensity for crystallites of arbitrary shape.

**Figure 6:**
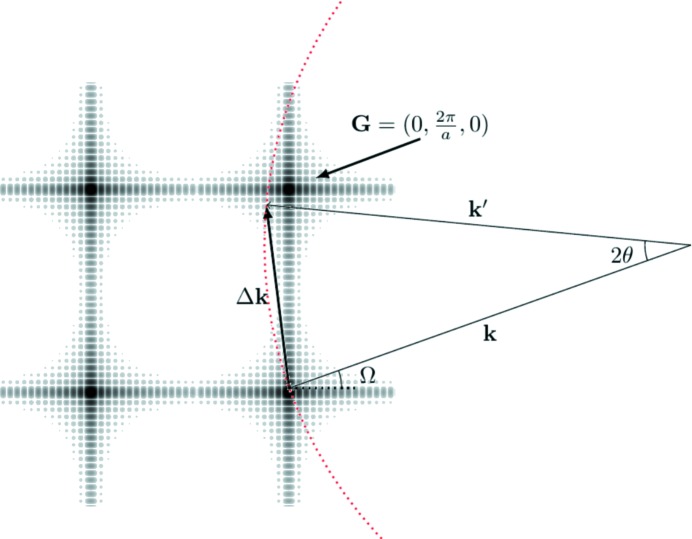
A plot of the intensity in reciprocal space predicted by equation (20) for a crystal with a cubic lattice, and of cubic shape, with the orientations of the crystal faces along the principal axes as described in the text. The incident and scattered X-rays and Ewald sphere corresponding to the setup in Fig. 1 ▸ are also shown.

A schematic plot of the distribution of intensity in the 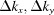 plane of reciprocal space given by equation (20) is shown in Fig. 6 ▸. We note that this figure is identical in form to Fig. 6-3(1) in the book edited by Ewald (1962 ▸).

For the sake of simplicity, consider now a crystal that is cubic in shape, such that 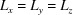. Fig. 6 ▸ shows the position 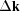 in reciprocal space corresponding to the scattering geometry of Fig. 1 ▸, where the cubic shaped crystal is set up for diffraction from the (010) planes, which also form a planar surface of the crystallite. Assuming that the number of planes is large, we can assume that for regions close to the Bragg condition 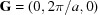 dominates in the sum in equation (20). From the geometrical construction in Fig. 6 ▸ we see that 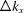 = 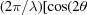 − 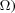 − 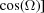, 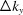 = 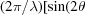 − 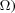 + 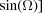, 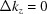 so that equation (20) becomes
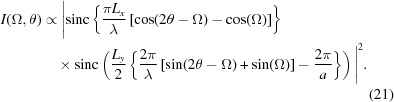



The intensity predicted by equation (21) is plotted in Fig. 2 ▸(*b*) for 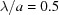 and 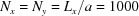. We note that the first term in equation (21) is identical to that in equation (5) of NTXRD [our equation (1)] for the case 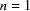, and hence still gives rise to a specular peak when 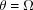. However, we no longer find a peak at exactly the Bragg condition as the angle Ω deviates from 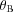. Nonetheless, we do find a peak at an angle 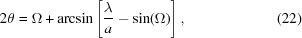
which for small deviations from the Bragg angle, 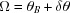, gives a peak in the intensity distribution when the detector is at an angle 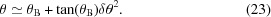
This ‘pseudo-Bragg’ peak is thus, for this particular case, a weak function of 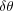, but we note that in contrast to NTXRD we do not find a diffraction peak exactly at the Bragg angle as the crystal is rotated away from the Bragg condition.

Recalling the error in the derivation of equation (1) [equation (5) of NTXRD] as outlined in §3, we note that, comparing our result with that of NTXRD, it can be seen that if the approximation that 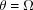 is made in the second term (but not the first) of equation (21), then the NTXRD formula, equation (1), is recovered.

We can also see from Fig. 6 ▸ that, for values of θ significantly larger than 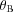, the Ewald sphere would intersect arms of the shape function that are associated with being centred on reciprocal-lattice vectors with 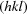 different from (010). We thus plot in Fig. 3 ▸(*b*) the intensity predicted by the full formula of equation (20), accounting for all reciprocal-lattice vectors with 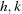 or *l* less than or equal to 2. As predicted, additional peaks seen around the detector positions of 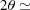 60° are visible, due to the Ewald sphere crossing the ‘arms’ of the shape transform lying between (110) and (120).

## 5. Geometrical interpretation and the general case   

A consideration of the geometry of the shape transform shown in Fig. 6 ▸ enables us to see why we observe a specular peak in intensity for this particular cubic shaped crystal, why this crystal also provides a peak in scattered intensity at an angle close to (but not exactly at) the Bragg angle when it is oriented away from the Bragg condition, and why in the general case NTXRD is incorrect.

The specular peak can be explained as follows. For an orthorhombic shaped crystal, with the facets cut as described thus far, the sinc functions associated with the shape transform form ‘arms’ of intensity parallel to the 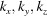 axes in reciprocal space. If the reflection in which we are interested has a reciprocal-lattice vector which lies along one of these arms then the arms of the shape function form a chord on the Ewald sphere (for the crystal cut as described here, any reciprocal-lattice vector in the family {*m*00} will meet this criterion). As can be seen from Fig. 6 ▸ the length of this chord will change as we vary Ω, but at a scattering angle 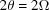 such that the reflection is always specular – a point to which we will return later.

**Figure 7:**
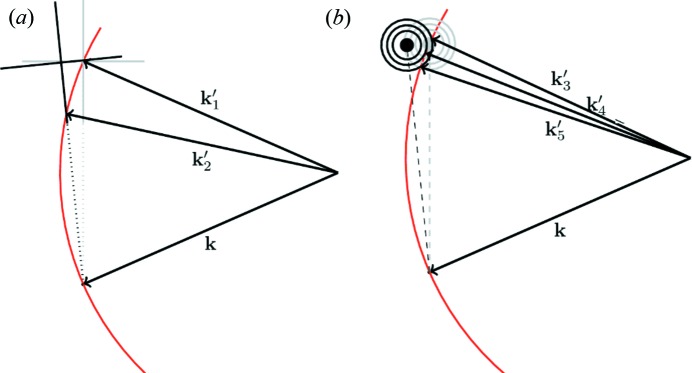
The maxima of the shape transforms for a cubic crystal (*a*) and a spherical crystal (*b*) are shown in schematic form for crystals rotated 6° from the Bragg condition for the (010) reflection. The 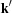 vectors indicate the intersection of the Ewald sphere and the shape transform maxima. The spherical shape transform has been truncated for clarity to only show the first three maxima.

The constructions in reciprocal space also allow us to see why we obtain some, albeit weak, scattering at close to (but not exactly at) the Bragg angle as this particular crystal is rotated for scattering associated with this particular reciprocal-lattice vector. Consider Fig. 7 ▸(*a*), where we show the shape transform for our cubic crystal at the Bragg condition and slightly rotated away from it. As an arm of the shape transform at the Bragg condition is perpendicular to the reciprocal-lattice vector, the position where the Ewald sphere crosses the arm of the shape transform is such that the angle of 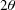 at such a point is initially a slowly varying function of the angle of rotation.

**Figure 8:**
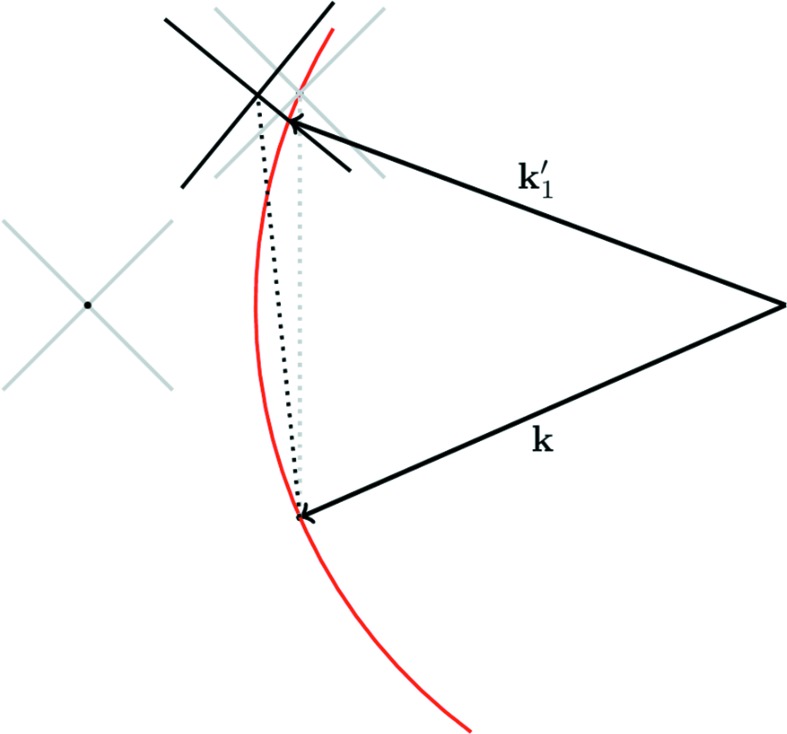
A schematic diagram of the shape transform in reciprocal space for a cubic crystal rotated such that radiation reflects from (110). Unlike for the (010) reflection, this does not exhibit a persistent pseudo-Bragg or specular peak in the intensity distribution.

We thus predict that diffraction from the same crystallites as considered to date (*i.e.* comprising a primitive cubic lattice, and cubic in shape with facets along the principal axes), but now diffracting from the (110) planes, will not exhibit peaks at the specular condition, or close to the Bragg condition when Ω deviates from 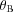. This can be seen from a sketch of the geometry in Fig. 8 ▸, where we can see that the arms of the shape transform are rotated 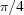 with respect to the reciprocal-lattice vector.

**Figure 9:**
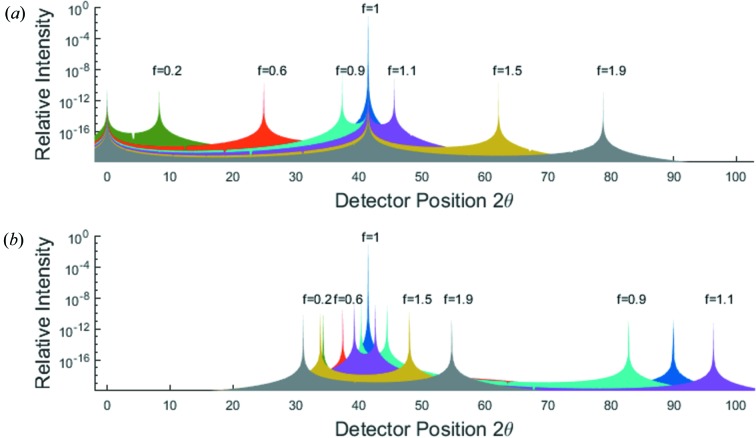
Intensity distribution contribution from the (110) reflection for the cubic crystal, using (*a*) the NTXRD method and (*b*) the method of equation (19). Note that while the two methods produced broadly similar results for the (010) reflection in Fig. 2 ▸, for this reflection they produce very different results.

This is indeed the case, and in Fig. 9 ▸(*b*) we show the results of the intensity predicted by equation (20) when diffracting from the (110) plane as a function of *f* as the crystal is rotated about the (001) axis. Once more we take 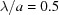, so that 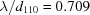 and 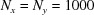. There is no peak in the diffracted intensity at the specular position, and diffraction associated with the original Bragg peak falls rapidly in intensity as *f* differs from 1.

This lies in stark contrast to the NTXRD result for the same reflection, seen in Fig. 9 ▸(*a*), which shows no qualitative difference to Fig. 2 ▸(*a*) besides the shifting of the Bragg angle.

Finally in this section we consider diffraction from a spherical crystal. We do so as within NTXRD it is claimed that ‘*the introduction of various shapes creates a different distribution of fringing, but the enhancement at* [*the Bragg angle*] *is still present* ’ – *i.e.* there is always an enhancement exactly at the Bragg angle, and spherical crystals are explicitly considered within NTXRD. As the Fourier transform of a solid uniform sphere can be written in terms of the half-integer Bessel functions of the first kind, for a spherical crystal of radius *R* (where we assume *R* is large compared with the lattice spacing), equation (19) can be written 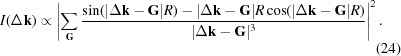



Thus, as can be seen from the reciprocal-space plot shown in Fig. 7 ▸, the spherical crystal shows a completely different pattern to that present in the cubic crystal previously discussed. Unlike the cubic shape transform’s distinct ‘arms’ which gave rise to the specular and slow-moving peaks, 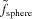 exhibits ‘ripples’, which cross the Ewald sphere a large number of times, giving rise to an extremely large number of residual peaks around a central maximum, the exact number of which changes rapidly as a function of crystal rotation.

**Figure 10:**
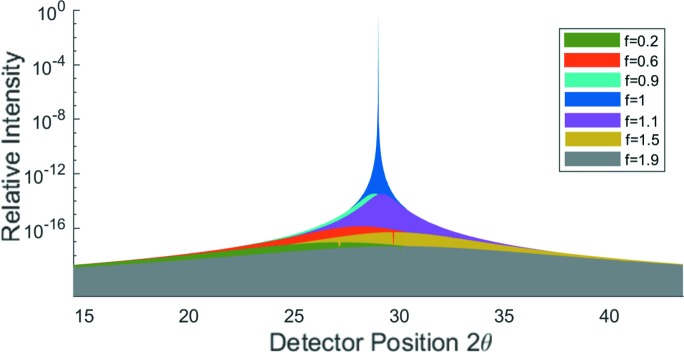
Intensity distribution for radiation incident at an angle 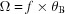 to the (010) plane of a spherical crystal, for a variety of values of *f*, as calculated from equation (24).

The intensity as a function of *f* for a spherical crystal is shown in Fig. 10 ▸ for 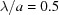 and 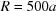. This figure is otherwise an exact replica of Fig. 2 ▸. Therefore, by the claims of NTXRD, we should see the same enhancement at the Bragg peak even when the Bragg condition is not satisfied, as well as specular reflections as observed in Fig. 2 ▸. However, no such features are observed, with only significant diffraction occurring at the Bragg condition, as expected.

We note that the fact that equation (24) describes the diffraction from spherical crystals has been recognized by other authors (Öztürk *et al.*, 2015 ▸).

## 6. Size broadening   

Thus, contrary to the claims made within NTXRD, crystals with different shapes do not have a persistent peak at the Bragg condition when Ω differs from 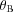. Indeed, the effects discussed thus far were already well understood in the earliest days of X-ray diffraction, and the widths of the Bragg peaks have been (within the approximations of this simple model) understood for of the order of a century. The Scherrer equation (Scherrer, 1918 ▸; Patterson, 1939 ▸) relates the peak width (full width at half-maximum, FWHM), 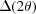, to the crystallite dimension *L* for nano-scale particles (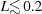
 µm): 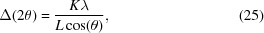
where *K* is the Scherrer constant, a function of crystal shape and which typically has a value of the order 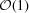.

**Figure 11:**
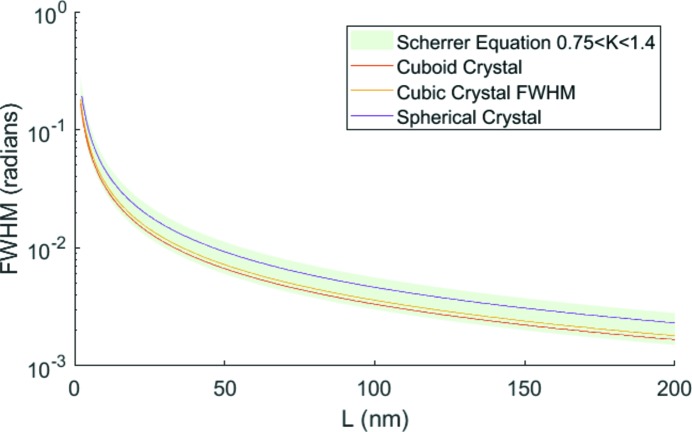
The FWHM of the diffracted intensity for a variety of crystal sizes and shapes calculated using equation (19). Each of these is accurately fitted by the Scherrer equation with *K* in the expected region.

Fig. 11 ▸ shows a simulation of the variation of the FWHM of the central peak with the crystallite dimension *L* of a variety of crystal shapes [cuboid (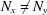), cubic (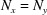) and spherical] calculated using equation (19). As a comparison, the region described by the Scherrer equation for 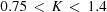 is also plotted, and it can be seen that all three of the simulations fall within this region.

More detailed analysis shows that each of these lines is accurately fitted by the Scherrer equation (within the nano-crystallite regime) with *K* values of 0.854, 0.898 and 1.156, respectively. Furthermore, that the finite size of crystals would give rise to diffraction away from the Bragg condition has also long been recognized (Bragg & Lipson, 1938 ▸).

## 7. Rotations about two axes   

As well as calculating the diffracted intensity for crystallites rotated about an axis perpendicular to the plane containing the source and detector, results are also given within NTXRD for simultaneous rotations of the crystallites through angles χ about a second axis, perpendicular to the first – being parallel to the *x* axis and passing through the crystal, as shown in Fig. 1 ▸. We consider once more the cubic shaped crystal, initially set up for Bragg diffraction from (010). We calculate the intensity at any given scattering angle as a function of Ω and χ from equation (20).

**Figure 12:**
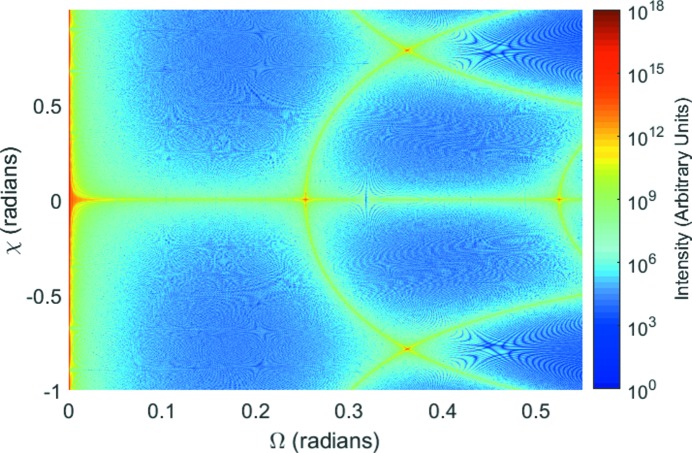
The intensity distribution for a two-axis rotation in the Bragg–Brentano geometry is shown for a cubic crystal of dimension 0.8 µm. Four Bragg peaks can be observed, as well as a series of arcs connecting them.

We consider diffraction in the Bragg–Brentano geometry (Bragg, 1921 ▸; Brentano, 1946 ▸), in which the detector is rotated along with the sample, such that 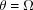. Once more we consider a cubic shaped crystal with a primitive cubic lattice and set 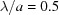. We show in Fig. 12 ▸ the intensity prediction as a function of Ω and χ.

The (010) peak in the scattering occurs, as expected, at the Bragg condition, 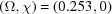, but we note that we can also observe a number of other Bragg peaks, with the (020) peak occurring at 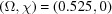, and finally the 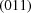 and 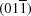 at 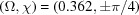, respectively. The scattered intensity that can be seen along 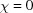 corresponds in form to the intensity as a function of 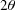 shown in Fig. 3 ▸ for 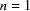.

In addition to these features, we also see an arc in the intensity distribution, passing through the Bragg condition, such that for values of Ω greater than that at the Bragg peak, for fixed Ω two further peaks are seen at finite χ. These peaks are easily understood in terms of the shape transform in reciprocal space. As the shape transform associated with the reciprocal-lattice point is rotated about the *x* axis, the arms of the shape transform lying along 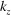 intersect the Ewald sphere for 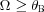; similar arcs can be observed elsewhere in the pattern, associated with the (020), (011) and (01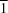) reflections, as expected from this model.

**Figure 13:**
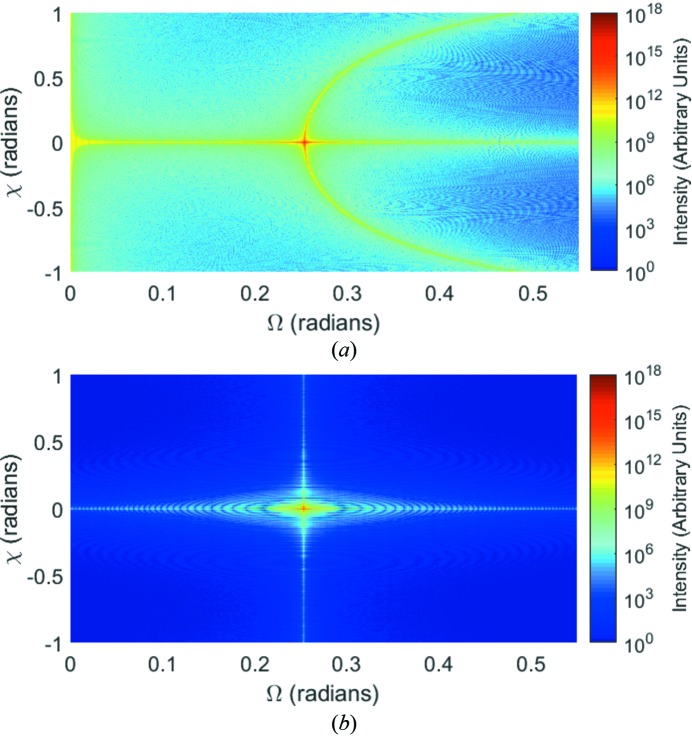
The intensity distribution for a two-axis rotation for a *fixed* detector is shown for a cubic crystal of dimension 0.8 µm using (*a*) the NTXRD formula and (*b*) equation (20).

At this juncture it would be useful for the reader to refer to Fig. 7 of NTXRD, which we have reproduced in Fig. 13 ▸(*a*). Our Fig. 12 ▸ resembles the NTXRD figure in a remarkable fashion, with the only major difference being that the NTXRD graph contains only contributions from (010), and hence does not display the other Bragg peaks. Some care should be taken in comparing the two plots, as we believe that Fewster may be assuming diffraction from the (111) plane of silicon, which has an f.c.c. (face-centred cubic) lattice – however the important point is that in NTXRD it is stated that Fig. 7 of that article is calculated for a *fixed* detector as Ω and χ are varied. We disagree that the sort of behaviour we observe in our Fig. 13 ▸(*a*), and in Fig. 7 of NTXRD, can correspond to the fixed detector geometry; it should only arise for the Bragg–Brentano geometry used to produce Fig. 12 ▸. Indeed, we plot in Fig. 13 ▸(*b*) the prediction of equation (20) for a fixed detector (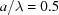, 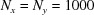). As expected from any conventional diffraction theory, under such conditions we then find only significant diffraction at the Bragg position itself.

It should be noted that the reverse does not hold true: the NTXRD prediction for a Bragg–Brentano detector does not resemble either Fig. 12 ▸ or Fig. 13 ▸(*b*).

## 8. Conclusion   

The effects that the finite size of crystals has on X-ray diffraction have been discussed and considered since soon after the foundation of the field. Within NTXRD mistakes are made in summing the phases of scattered X-rays from a crystal with an orthorhombic shape, which lead to the incorrect conclusion that such crystals always have some peak in scattering at the Bragg condition. It is also claimed that this result holds for crystals of a general shape. As we have shown, these conclusions are in error, and the effects that the shape and finite size of crystals have on the diffraction pattern are well described by conventional diffraction theory.

Whilst a study of finite crystallite size effects will no doubt continue to be of importance in relating experimental and computed diffraction profiles, and the experimental data presented in Fewster (2014 ▸) and Fewster (2016 ▸) are no doubt worthy of further study, the specific claim made within NTXRD that simple theory predicts a peak in the scattered intensity to occur exactly at the Bragg condition when small crystallites are rotated away from that condition is false.
